# Research on Parkinson’s disease immunotherapy: a bibliometric analysis via multiple databases

**DOI:** 10.3389/fimmu.2025.1659848

**Published:** 2025-10-08

**Authors:** Xinxing Fei, Shiqi Wang, Jiayi Song, Caihong Cao, Yaqian Gao, Yue Hu

**Affiliations:** ^1^ Department of Psychiatry, Chengdu Eighth People's Hospital (Geriatric Hospital of Chengdu Medical College), Chengdu, China; ^2^ Rehabilitation Medicine Center and Institute of Rehabilitation Medicine, West China Hospital, Sichuan University, Chengdu, Sichuan, China; ^3^ Key Laboratory of Rehabilitation Medicine in Sichuan Province, Chengdu, Sichuan, China; ^4^ Department of Rehabilitation Medicine, The Affiliated Hospital of Southwest Medical University, Luzhou, China; ^5^ Rehabilitation Medicine and Engineering Key Laboratory of Luzhou, Luzhou, China; ^6^ Department of Rehabilitation Medicine, The First Affiliated Hospital of Chengdu Medical College, Chengdu, China; ^7^ Centro Studi e Ricerche in Neuroscienze Cognitive, Dipartimento di Psicologia, Alma Mater Studiorum – Università di Bologna, Cesena, Italy

**Keywords:** Parkinson’s disease, immunotherapy, bibliometrics, hotspots, frontiers

## Abstract

**Background:**

Parkinson’s disease (PD) is a neurodegenerative disorder involving degeneration of dopaminergic neurons in the substantia nigra, abnormal aggregation of α-synuclein, and neuroinflammatory response. Although research on PD immunotherapy is advancing rapidly, bibliometric analysis in this field remains underdeveloped.

**Methods:**

Literature related to “Parkinson’s disease” and “immunotherapy” from the Web of Science Core Collection and Scopus was used for data merging and bibliometric analysis via Bibliometrix. The characteristics of the relevant clinical trials in this field retrieved from the PubMed database were summarized, and the study protocols were traced back through the trials registry website.

**Results:**

After merging the two databases, a total of 890 documents from 488 sources were covered. A total of 3,804 researchers from 1,483 institutions in 63 countries published research in this field. Authors, institutions, and countries/regions were classified into 5, 12, and 13 clusters, respectively. Keywords such as “Parkinson’s disease”, “immunotherapy”, and “alpha-synuclein” were frequently used. The maps of keyword co-occurrence and clusters revealed the generation of two clusters. A total of 8 clinical trials were searched and included. These trials focused on active immunotherapy and targeted antibodies, involving both healthy volunteers and patients with PD.

**Conclusions:**

The field of PD immunotherapy has vigorous development potential. The current research focus in this field is concentrated on analyzing pathological mechanisms and innovating treatment strategies. Precise immunological intervention techniques are frontiers in this field. In the future, large-scale randomized controlled trials should be conducted to enhance the clinical translation efficiency of immunotherapy.

## Introduction

Parkinson’s disease (PD) is a neurodegenerative disorder characterized by movement disorders and non-motor symptoms. The main pathological changes include degeneration of dopaminergic neurons in the substantia nigra, abnormal aggregation of α-synuclein, and neuroinflammatory response (1). Globally, the number of patients with PD continues to increase as the aging population progresses. However, the underlying mechanism of PD has not been fully elucidated, and current treatment methods primarily focus on alleviating symptoms, rather than preventing disease progression (2, 3). In recent years, immunotherapy has become a research hotspot in the field of PD treatment due to its potential to regulate the immune microenvironment and target the clearance of pathological markers (4). Immunotherapy, by regulating both innate and adaptive immune responses, may slow down the aggregation of α-synuclein, inhibit neuroinflammation, and thereby delay the progression of the disease, providing a novel therapeutic strategy for PD.

Currently, the application forms of PD immunotherapy are diverse, primarily including passive immunity, such as monoclonal antibodies targeting α-synuclein, active immunization vaccines, and innate immune regulators (5, 6). For instance, monoclonal antibodies against α-synuclein have entered the clinical trial stage, aiming to reduce neuronal damage by neutralizing toxic protein aggregates (7, 8). However, immunotherapy still faces numerous challenges, including the efficiency of penetrating the blood-brain barrier, the off-target effects caused by the insufficient specificity of the targets, and the balance issue between the complexity of the immune system and the heterogeneity of the disease (9). These bottlenecks have restricted its clinical application and prompted the need for a systematic review of the research progress and shortcomings.

Notably, although clinical and basic research on PD immunotherapy is advancing rapidly, bibliometric analysis in this field remains lacking. Through the quantitative analysis of literature data, bibliometric analysis can reveal the research trends, hotspots, and knowledge structure of a field, providing a comprehensive perspective for the development of the discipline (10). The development of bibliometric visualization tools has also provided significant assistance to the hot topics in the field of visualization (11). However, many bibliometric studies mostly rely on a single database, neglecting the advantages of a combined database search, which may result in incomplete data coverage (12). Furthermore, most studies focus on quantitative indicators, such as the number of publications and author cooperation networks, while paying insufficient attention to the integrated analysis of key clinical elements, including clinical trial design, efficacy evaluation, and safety (13).The tendency to emphasize data statistics while neglecting clinical relevance weakens the support role of bibliometrics in actual clinical decision-making, highlighting the need for cross-database integration and clinically oriented analysis.

To summarize, this study aims to conduct a systematic assessment of the current research status and the evolution trajectory of the PD immunotherapy field by integrating resources from multiple databases and utilizing visualization tools. By constructing multidimensional indicators, such as cooperation networks, research hotspots, and clinical trial progress, and combining quantitative analysis with clinical evidence summaries, we further reveal the scientific issues and technical bottlenecks that remain unresolved in the field of PD immunotherapy.

## Methods

### Data search

To explore the research landscape on PD and immunotherapy, we conducted a systematic literature search in three databases, including the Web of Science Core Collection (WoSCC), Scopus, and PubMed. The search aimed to identify relevant scientific publications for bibliometric analysis and clinical trials related to PD immunotherapy. We searched original articles and review papers from WoSCC and Scopus for bibliometric analysis, focusing on clinical trials from PubMed, including both interventional and observational studies.

The search strategies were specifically designed for each database and are presented in full detail in **Supplementary Tables S1**-**S3**, which include the verbatim search strings used. A combination of free words and subject terms was used to enhance the sensitivity and specificity of the search. All subject terms were derived from the Medical Subject Headings (MeSH) database. No language restrictions were applied during the initial search to minimize potential bias from excluding non-English publications. To ensure data integrity and avoid double-counting, duplicate entries were identified and removed through a combination of automated deduplication and manual inspection. The last search date for all the databases was July 2nd, 2025. The search, de-duplication, and initial data organization were independently conducted by two researchers, with any discrepancies resolved through discussion and collaboration.

### Data analysis

Bibliometrix, as a comprehensive analysis toolkit based on the R language, has become an essential support in the field of research due to its multi-dimensional analysis capabilities and visualization features (14). This tool supports cross-database data integration and can uniformly handle the metadata of multiple literature sources. In our study, we used Bibliometrix (Version 5.0) to merge data from the WoSCC and Scopus databases, and manually checked and recorded the key bibliometric indicators for each database.

The Overview module was used to compare the data differences between the databases. The Sources, Authors, and Cooperation Analysis modules were used to conduct a visual analysis of the merged data, and the Keywords module was used to analyze the research frontiers and characteristics of this field (15). Of note, international cooperation articles were determined based on whether a publication had authors from multiple countries/regions. The rate of international cooperation was calculated by dividing the number of these articles by the total number of papers in the field. The division of countries/regions is determined by using the institutional information in the database (WoSCC and Scopus) and is completed through processing with Bibliometrix.

In addition, based on the PubMed search results, we further summarized the characteristics of relevant clinical trials, including study design, interventions, and effects, to provide complementary insights into the translational and clinical aspects of PD immunotherapy research. Notably, only studies that explicitly employ immunotherapy in their intervention measures could be included. Furthermore, based on the clinical trial registration number (if applicable), we traced the original study protocol back through the trial registration website, such as ClinicalTrials.gov and the EU Clinical Trials Register (EU CTR), to strive for standardization and consistency in the research plan and results.

### Author, institution, and keyword standardization

To ensure the comparability of the research data and the reliability of the analytical results, this study conducted systematic standardization of author information, institutions, and keywords in the original bibliographic dataset. In the raw data, author names exhibited various spelling variations, inconsistencies in capitalization, and formatting differences, such as “Wei Zhang,” “Zhang, W.”, and “W. Zhang”. After data integration, Bibliometrix was used to standardize author names into a unified format of ”surname + initial”, such as “Zhang W,” to facilitate statistical analysis and network construction. However, as unique author identifiers such as ORCID were not used, some author nodes may still have experienced minor duplication or incorrect merging. Therefore, the related results and conclusions are primarily based on macro-level cooperation patterns, rather than precise individual-level relationships.

For institutions, multiple variations in naming conventions, abbreviations, and aliases existed for the same organization. To improve the consistency of the institutional cooperation network, we applied the ”Affiliation Name Disambiguation” function in Bibliometrix to identify and merge variants under the same institutional entity. It should be noted that certain institutions, although administratively or organizationally part of the same university system, such as Harvard University and Harvard Medical School, may still appear as distinct entities in bibliometric data and network analysis—even when sharing the same institutional identifier such as ROR. Consistent with approaches used in other studies, these institutions were treated as independent nodes in our analysis to more accurately reflect their actual roles in scientific cooperation and knowledge production.

Due to the inconsistencies in capitalization, singular/plural forms, and formatting of the keywords, we manually standardized the synonymous/near-synonymous variants in the original data. This included merging “Parkinson’s disease” and “Parkinson disease” into “parkinson’s disease,” and combining “alpha-synuclein” and “alpha synuclein” into “alpha-synuclein.” Moreover, we deleted the general terms that were not directly related to the analysis of research hotspots, such as “review,” “article,”“ priority journal,” “unclassified drug,” “unindexed drug,” “animal,” “nonhuman,” and “human” to avoid diluting the relevance of the core topic.

### Sensitivity analysis of citation indicators

Citations are commonly used as an indicator of the impact of scientific research. However, their values can be influenced by factors such as paper type, publication year, research field, and the presence of extremely highly cited papers, which may introduce bias in the calculation of average citation performance at the country or regional level. To ensure the reliability of the core findings related to the citation performance of countries/regions in this study, we conducted the following sensitivity analyses (1): Exclusion of review articles: We first excluded publications classified as “Review” (based on document type), and recalculated the average article citations for each country/region, to examine whether the core conclusions remained valid (2). Exclusion of extremely highly cited papers: A small number of papers may receive citation counts far above the average. To minimize the impact of such outliers on the statistical results, we excluded the top 5% of papers with the highest citation counts from the data and recalculated the average article citations (3). Citation analysis by publication year cohorts: Given the well-established time accumulation effect in citation performance, we divided the publication years into distinct time cohorts (1980–2005, 2006–2015, and 2016–2025), based on both year and publication volume. For each time cohort, we calculated the average number of article citations by country during the respective period.

### Parameters setting

The parameter settings of Bibliometrix for different visualization networks are as follows (1): Cooperation analysis: Network Layout: Automatic layout; Clustering Algorithm: Walktrap; Normalization: association; Number of Nodes: 50; Repulsion Force: 0.1; Remove Isolated Nodes: Yes; Minimum Number of Edges: 1 (2). WordCloud: Word occurrence by: Frequency; Shape: Circle; Font type: Impact; Text colors: Random Dark; Font size: 1.3; Ellipticity: 0.65; Padding: 1; Rotate: 0 (3). Keyword co-occurrence analysis: Network Layout: Automatic layout; Clustering Algorithm: Walktrap; Normalization: association; Number of Nodes: 50; Repulsion Force: 0.1; Remove Isolated Nodes: Yes; Minimum Number of Edges: 2 (4). Thematic Evolution: Number of Words: 250; Min Cluster Frequency (per thousand docs): 5; Weight index: Inclusion Index weighted by Word-Occurrences; Min Weight Index: 0.1; Label size: 0.2; Number of Labels (for each cluster): 3; Clustering Algorithm: Walktrap. Time Slices: Number of Cutting Points: 4; Cutting Year 1: 2005; Cutting Year 2: 2010; Cutting Year 3: 2015; Cutting Year 4: 2020 (5). Trend topics: Word Minimum Frequency: 5; Number of Words per Year: 3.

## Results

### Overview

**Figure 1:**
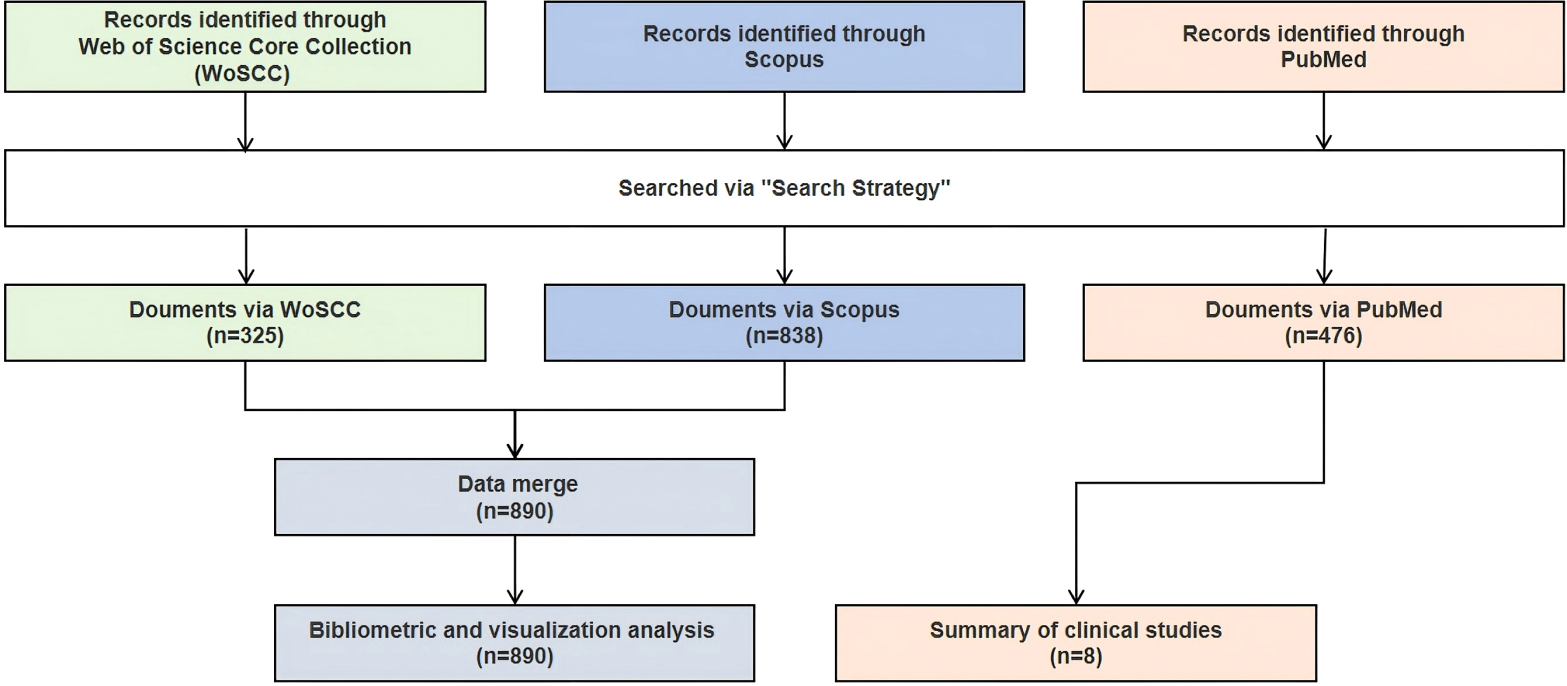
Flow chart.

In the field of PD immunotherapy, WoSCC covered 325 documents from 190 sources, while Scopus covered 838 documents from 454 sources. After merging the two databases (WoSCC + Scopus), a total of 890 documents from 488 sources were covered (**Figure 1**). The annual growth rate of the combined database was 8.88%, and the average citation intensity reached 69.11 times per paper, which was higher than that of individual databases. Moreover, the combined database contained 9,648 keywords, and the international co-authorship ratio and the average number of authors per document were also within the range of WoSCC and Scopus (**Table 1**). Through the integration of complementary data resources, the breadth of literature coverage and the depth of knowledge association have been significantly enhanced.

**Table 1:** Overview of PD immunotherapy in different databases.

Description	Results (WoSCC)	Results (scopus)	Results (WoSCC + scopus)
Timespan	1992:2025	1980:2025	1980:2025
Sources (Journals, Books, etc.)	190	454	488
Documents	325	838	890
Annual Growth Rate %	9.15	8.66	8.88
Average citations per document	41.02	67.55	69.11
Document contents			
Keywords Plus	1164	10004	9648
Author’s Keywords	812	2000	2086
Authors			
Authors	1716	3693	3804
Authors of single-authored documents	16	80	80
Authors cooperation			
Single-authored documents	17	86	86
Co-Authors per document	6.62	5.32	5.48
International co-authorships%*	33.23 (108/325)	26.49 (222/838)	26.74 (238/890)

*International co-authorship is defined as the proportion of publications in the field that involve authors from more than one country. PD, Parkinson’s disease; WoSCC, Web of Science Core Collection.

**Figure 2:**
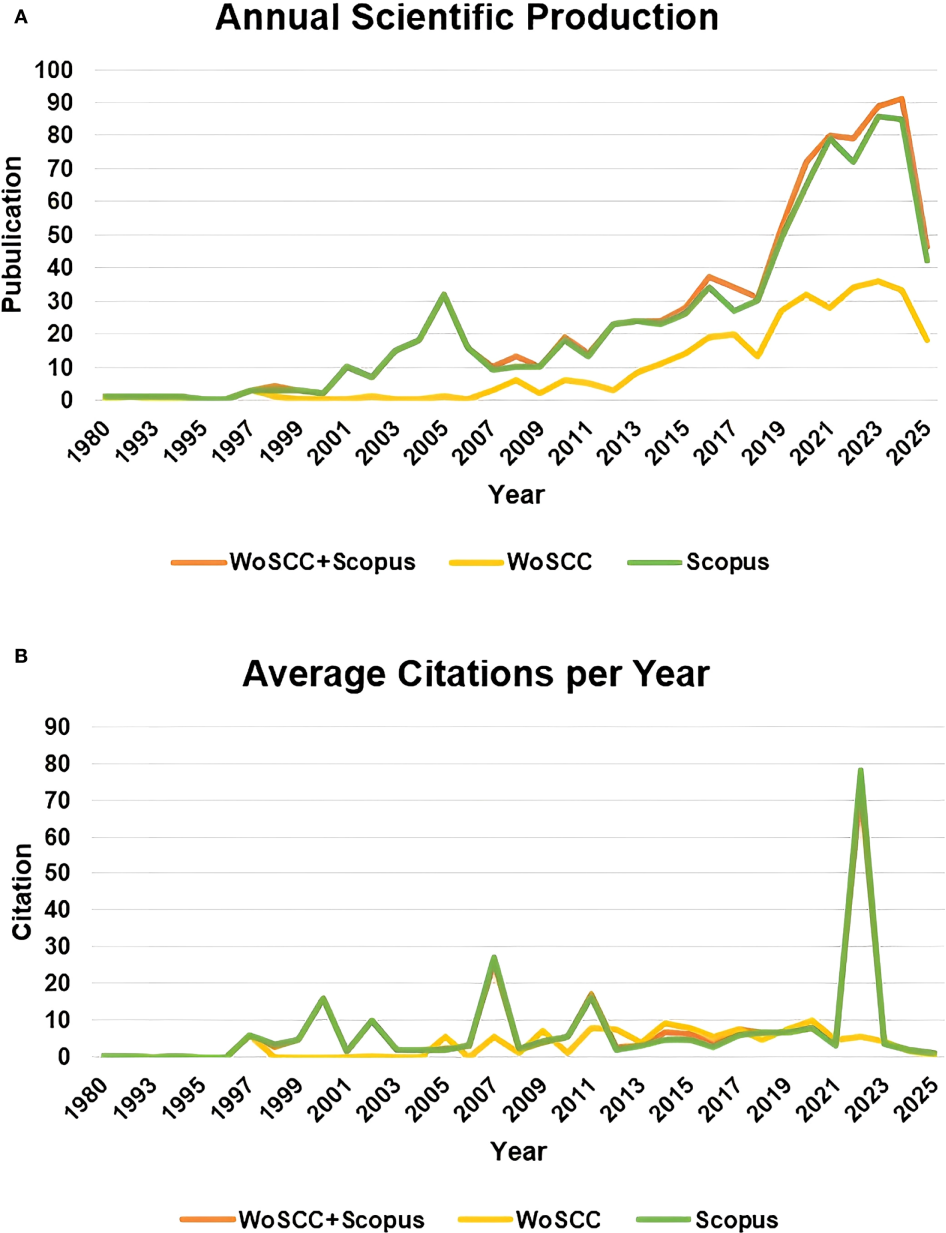
Publications and citations. **(A)** Annual scientific production. **(B)** Average citations per Year.

From the perspective of the publication and citation trends, the publication volume of the combined database showed explosive growth in key years such as 2019 and 2022, covering the entire period from 1980 to 2025, with the average annual publication volume significantly higher than that of the single database (**Figure 2A**); in terms of citation intensity, the combined database reached its peak average citation frequency in 2007 (25.69 times per documents) and 2022 (73.03 times per documents), with the fluctuation range far exceeding that of the single database (**Figure 2B**).

The above results reflected the integration advantage of multi-database analysis in integrating exceptionally high-impact papers, enabling a more comprehensive support for the construction and evolution path analysis of the knowledge map in a specific field. Therefore, our subsequent results were based on the thorough analysis of the merged data from the two databases.

### Sources

**Figure 3:**
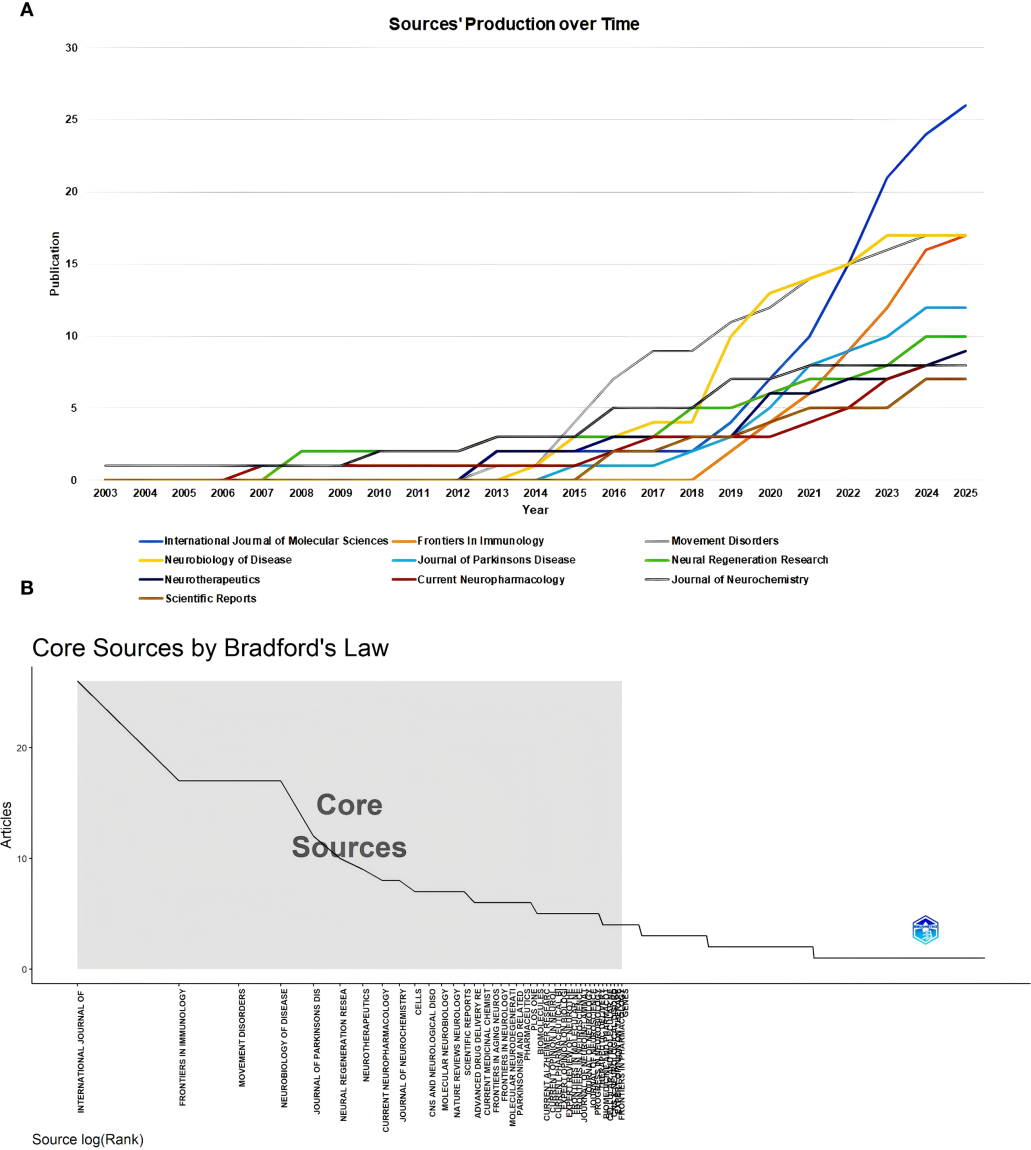
Source analysis. **(A)** Sources’ production over Time. **(B)** Core sources by Bradford’s Law.

A total of 488 sources published all the documents in this field. The top 10 most popular sources have been seen in an increasing number of publications year by year (**Figure 3A**). As of now, the journal with the highest number of publications was the *International Journal of Molecular Sciences*, the journal with the highest impact factor (IF) was *Movement Disorders*, and the journal with the highest H-index was *Neurobiology of Diseases* (**Table 2**). We further determined the core sources in the field of immunotherapy for 41 sources using Bradford’s Law (**Figure 3B**).

**Table 2:** Top 10 popular sources in this field.

Source	Publication	IF	H-index	G-index	M-index	Citation	Year*
*International Journal of Molecular Sciences*	26	4.9	13	26	1	903	2013
*Neurobiology of Disease*	17	5.6	14	17	1.17	889	2014
*Movement Disorders*	17	7.6	13	17	1	909	2013
*Frontiers In Immunology*	17	5.9	5	12	0.71	154	2019
*Journal of Parkinsons Disease*	12	5	10	12	0.91	500	2015
*Neural Regeneration Research*	10	6.7	5	10	0.28	121	2008
*Neurotherapeutics*	9	6.9	6	9	0.46	225	2013
*Journal of Neurochemistry*	8	4	5	8	0.22	306	2003
*Current Neuropharmacology*	8	5.3	4	8	0.21	75	2007
*Scientific Reports*	7	3.9	6	7	0.6	194	2016

*Year of first publication. IF, impact factor.

### Authors, institutions, and countries/regions

**Figure 4:**
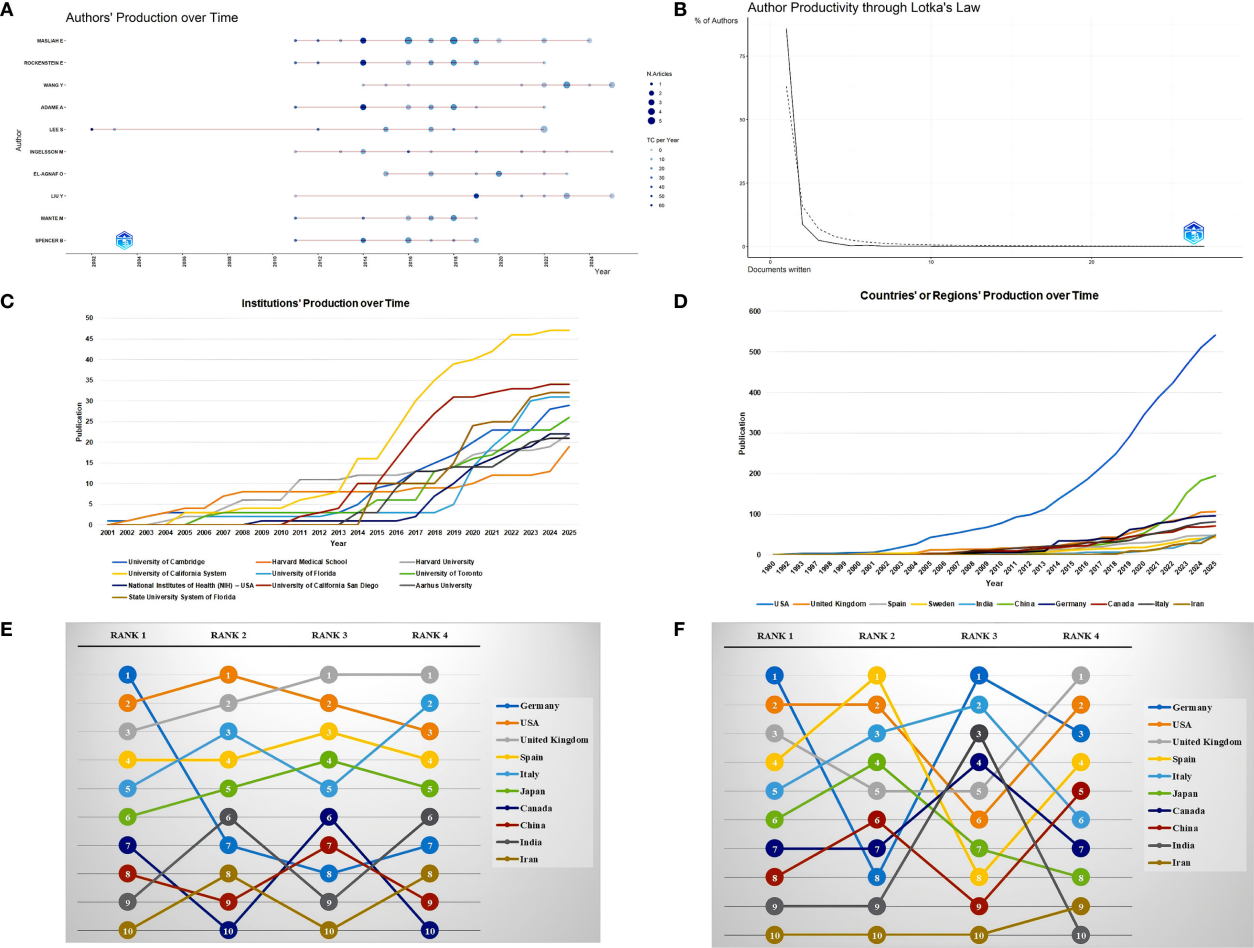
Author, institution, and country/region analysis. **(A)** Authors’ production over time. **(B)** Author Productivity through Lotka’s Law. **(C)** Institutions’ production over time. **(D)** Countries’ or regions’ production over time. **(E)** Sensitivity analysis of Countries/regions citation indicators. All rankings are based on the calculation of average article citation. RANK1: all articles; RANK2: no reviews; RANK3: no top 5% highly cited; RANK4: no reviews and top 5% highly cited. **(F)** Country/region citation analysis grouped by publication year. All rankings are based on the calculation of average article citation. RANK1: all articles; RANK2: articles published in 1980-2005; RANK3: articles published in 2006-2015; RANK4: articles published in 2016-2025.

A total of 3,804 researchers from 1,483 institutions in 63 countries published research in this field. Masliah E ranked first with 27 publications and an H-index of 18, followed by Rockenstein E (15/14) and Adame A (13/13). Although the publication volume of some authors, such as Gendelman H and Bergstrom J, was not high, they had relatively small differences between their publication volume and H-index, which indirectly reflects the stability of their output scale and the influence of their achievements (**Table 3**, **Figure 4A**). Moreover, a large number of low-productivity authors dominate, with 85.96% publishing only one paper. The scale of high-productivity authors has significantly shrunk. In particular, only 19 people have published more than five publications, which deviates from the exponential decay pattern of Lotka’s law (**Figure 4B**).

**Table 3:** Top 10 popular authors in this field.

Author	ORCID	Publication	H-index	G-index	M-index	Citation	Year*
Masliah E	0000-0002-2117-5569	27	18	27	1.20	1999	2011
Rockenstein E	0000-0001-8905-2859	15	14	15	0.93	1674	2011
Adame A	–	13	13	13	0.87	1303	2011
Mante M	0000-0002-3300-3377	10	10	10	0.67	1028	2011
Spencer B	–	10	10	10	0.67	1158	2011
Ingelsson M	0000-0001-5466-8370	11	9	11	0.60	697	2011
Lee S	–	12	9	12	0.38	2330	2002
El-Agnaf O	0000-0002-6850-8084	10	8	10	0.73	434	2015
Gendelman H	–	9	8	9	0.50	282	2010
Bergström J	–	8	7	8	0.47	378	2011

*Year of first publication.

The University of California System ranked first with 47 publications, followed by the University of California San Diego (34) and the State University System of Florida (32). The top 10 most popular institutions consistently produced research outputs each year (**Table 4**, **Figure 4C**). We calculated the number of published papers for each country/region based on the country of the “corresponding author” (**Table 5**, **Figure 4D**). The results showed that the USA topped the list with 232 publications, followed by China with 108 publications. The United Kingdom (57), Italy (49), and India (45) ranked third to fifth, respectively.

**Table 4:** Top 10 popular institutions in this field.

Institution	ROR ID	Publication
University of California System	00pjdza24	47
University of California San Diego	0168r3w48	34
State University System of Florida	05sqd3t97	32
University of Florida	02y3ad647	31
University of Cambridge	013meh722	29
University of Toronto	03dbr7087	26
Harvard University	03vek6s52	22
National Institutes of Health (NIH) - USA	01cwqze88	22
Aarhus University	01aj84f44	21
Harvard Medical School	03vek6s52	19
National Institute on Aging (NIA)	049v75w11	18

**Table 5:** Top 10 popular countries/regions in this field.

Countries/regions	Publication	Publication % (Publication/Total)	Citation	Average article citation	SCP	MCP*	MCP % (MCP/Publication)
USA	232	26.07 (232/890)	13124	56.6	189	43	18.53 (43/232)
China	108	12.13 (108/890)	1624	15.0	87	21	19.44(21/108)
United Kingdom	57	6.40 (57/890)	2808	49.3	34	23	40.35 (23/57)
Italy	49	5.51 (49/890)	1638	33.4	36	13	26.53 (13/49)
India	45	5.06 (45/890)	673	15	32	13	28.89 (13/45)
Germany	29	3.26 (29/890)	5202	179.4	17	12	41.38 (12/29)
Canada	27	3.03 (27/890)	484	17.9	15	12	44.44 (12/27)
Spain	22	2.47 (22/890)	892	40.5	13	9	40.91 (9/22)
Iran	18	2.02 (18/890)	172	9.6	10	8	44.44 (8/18)
Japan	18	2.02 (18/890)	350	19.4	13	5	27.78 (5/18)

*MCP values are based on the corresponding author assignment, not on all co-authors’ countries/regions. MCP, multi-country authors; SCP, single-country authors.

We further compared the performance of citation indicators among the top 10 countries/regions with the highest publication volumes. Germany ranked first with an average article citation of 179.4, followed by the USA (56.6) and the United Kingdom (49.3), which ranked second and third, respectively. China, despite having a relatively high publication volume, ranked relatively low in terms of citation data. After excluding review articles, Germany’s average article citations dropped sharply from first (179.4) to seventh (16.0), the United Kingdom (27.5) jumped to second, and the USA (47.4) remained first, showing that these two countries still had relatively high average article citations even when only original research was considered. After excluding the top 5% of papers with the highest citation counts, Germany’s average article citations further decreased to 12.5, dropping to eighth. In contrast, the United Kingdom (29.3) jumped to first, and the USA (28.6) ranked third. After excluding both review articles and the top 5% of papers with the highest citation counts simultaneously, Germany’s average article citations remained at 16.0, still ranking seventh, the United Kingdom (27.5) ranked first again, and the USA (20.9) ranked third (**Supplementary Table S4**; **Figure 4E**). In addition, we calculated and ranked the average article citations of papers from each country across different time periods, categorized by publication year cohort. Germany had a low ranking (Rank = 8) in the early stage, but its average article citations improved significantly in the mid-term (Rank = 1) and then declined somewhat in the recent period (Rank = 3). The USA maintained a leading position for a long time, but its ranking fluctuated in different stages. The United Kingdom rose steadily from fifth in the early stage and rose to first in the past decade (**Supplementary Table S5**; **Figure 4F**).

### Cooperation analysis

**Figure 5:**
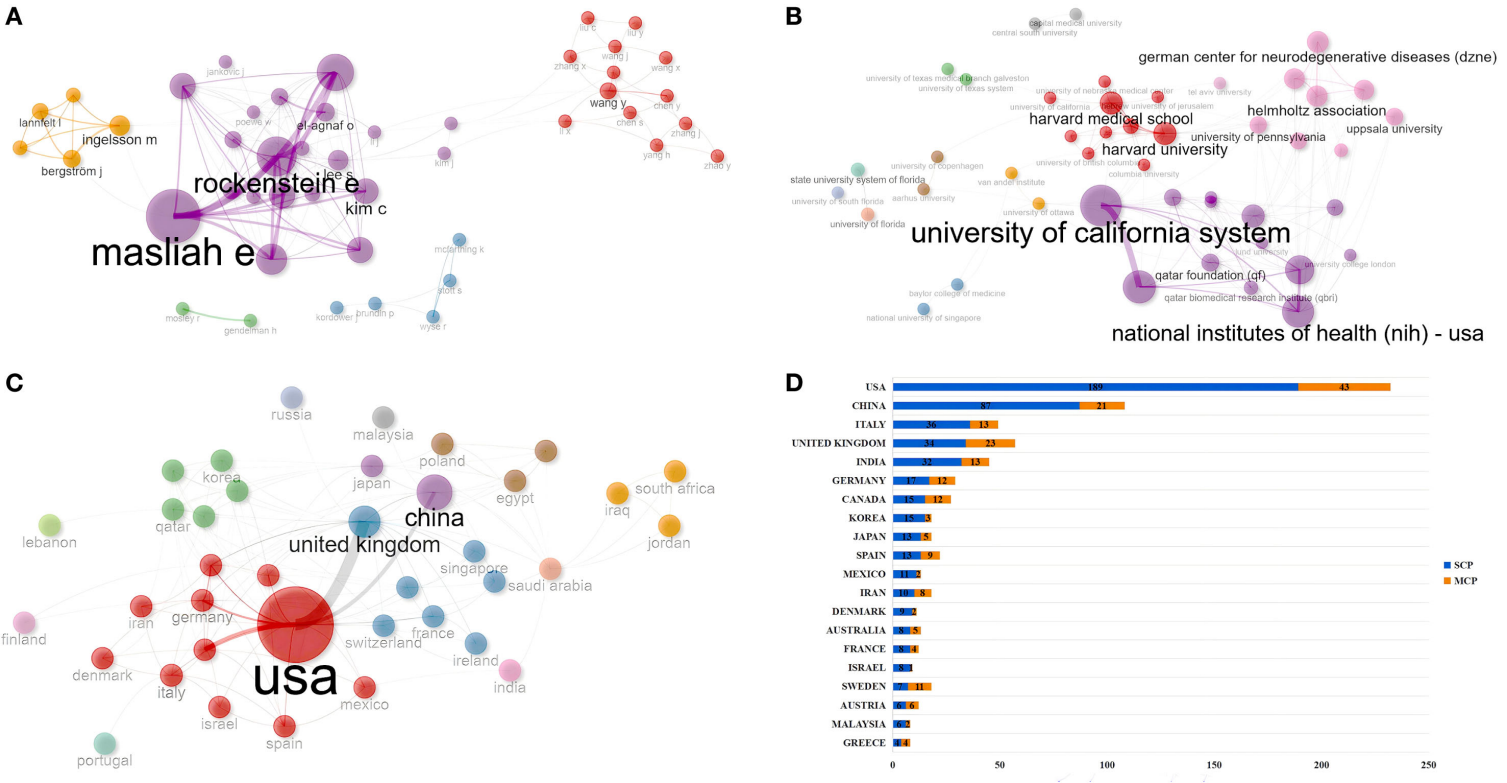
Cooperation analysis. **(A)** Map of author co-occurrence. **(B)** Map of institution co-occurrence. **(C)** Map of country/region co-occurrence. **(D)** The cooperation model between countries/regions. MCP, multi-country authors; SCP, single-country authors.

Cooperation analysis can reveal the cooperative network structure of each individual (author, institution, or country/region) in this field, promote interdisciplinary and international cooperation, optimize research management, and drive innovation. In the field of PD immunotherapy, the authors were classified into 5 clusters (**Figure 5A**). Masliah E might lead the core cooperation with Lee S, El-Agnaf O, and Vaikath N. Lee S formed a close sub-network with Kim C, Rissman R (**Supplementary Table S5**). In terms of institutional cooperation, institutions were classified into 12 clusters (**Figure 5B**). Harvard Medical School was the core hub, connecting institutions such as Harvard University and Columbia University; the University of California System served as a cross-regional bridge, linking institutions such as the National Institutes of Health (NIH) - USA and the University of Oxford (**Supplementary Table S6**). In terms of countries/regions cooperation, the countries/regions were classified into 13 clusters (**Figure 5C**). The USA served as the core hub, connecting countries and regions with high influence, such as Germany, Italy, and Canada. The United Kingdom led cross-regional cooperation, coordinating with countries and regions like France and Austria. Qatar acted as a bridge connecting China and Australia, but South Africa and Iraq, among others, had a betweenness of 0, indicating weak internal connections. China formed a regional cooperation core with Japan (**Supplementary Table S7**). We further analyzed the cooperation situation among the countries/regions where the “corresponding authors” of each publication were located. Among the countries with the highest number of publications, the USA (18.53%) and China (19.44%) had relatively low proportions of international cooperation (**Figure 5D**).

### Keywords

**Figure 6:**
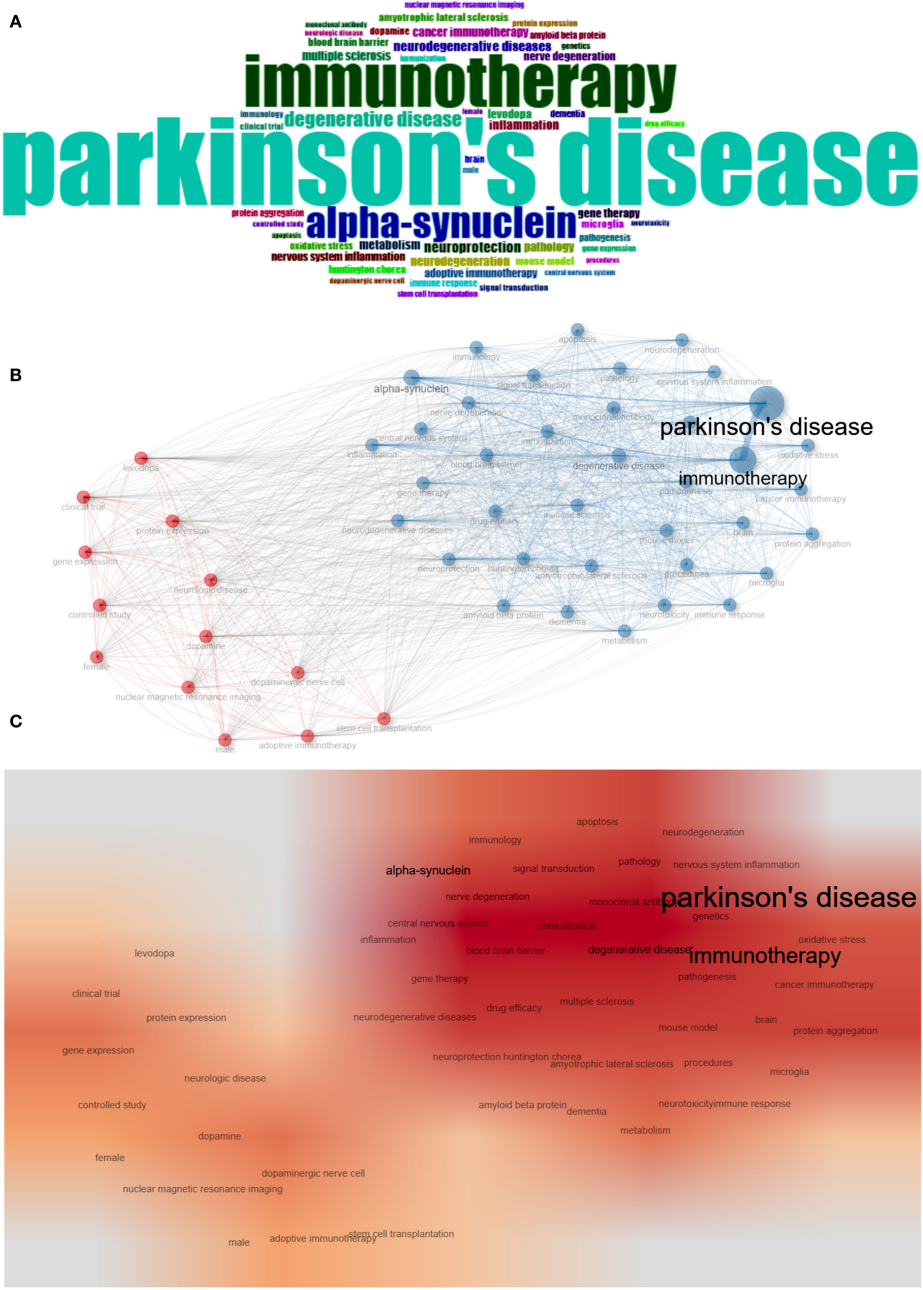
Keyword analysis. **(A)** WordCloud. **(B)** Map of keyword co-occurrence. **(C)** Heat map of keyword co-occurrence.

Keyword analysis plays a crucial role in identifying hotspots in the field and predicting the research frontiers. Keywords such as “Parkinson’s disease,” (835) “immunotherapy,” (495) and “alpha-synuclein” (305), were frequently used in this field (**Figure 6A**, **Table 6**). The maps of keyword co-occurrence and clusters revealed the generation of two main clusters (**Figure 6B**). Cluster 1, comprising largely methodological, experimental, and clinical keywords, functioned as a foundational domain centered on research design, diagnostic tools, and therapeutic interventions. This cluster was anchored by terms such as “clinical trial”, “controlled study”, and “dopamine”, all of which exhibited moderate centrality values. Other notable keywords included “levodopa”, “dopaminergic nerve cell” , “protein expression”, and “gene expression”. In contrast, Cluster 2 emerged as the dominant thematic hub, integrating concepts related to neurodegenerative pathology, therapeutic innovation, and disease mechanisms, with significantly higher betweenness centrality among its key nodes. The most influential and structurally central keyword in the entire network was “Parkinson’s disease”, which acted as the primary node bridging multiple sub-themes and anchoring the cluster’s conceptual identity. Other highly central terms within this cluster included “immunotherapy”, “neurodegeneration”, “neurodegenerative diseases”, “alpha-synuclein”, and “neuroprotection”. These keywords reflect the high emphasis on the mechanistic understanding of neurodegenerative processes, particularly those related to protein misfolding, inflammation, oxidative stress, and neuronal death, as well as covering emerging therapeutic strategies such as immunotherapy, gene therapy, and neuroprotective interventions (**Supplementary Table S8**). The heat map also indirectly indicated that Cluster 2 was currently the research hotspot in this field (**Figure 6C**).

**Table 6:** Top 20 keywords in the field.

Keywords	Frequency
Parkinson’s disease	835
immunotherapy	495
alpha-synuclein	305
degenerative disease	149
neuroprotection	108
neurodegenerative diseases	100
inflammation	95
metabolism	94
neurodegeneration	93
pathology	91
cancer immunotherapy	90
multiple sclerosis	89
levodopa	87
gene therapy	86
nerve degeneration	85
adoptive immunotherapy	82
nervous system inflammation	82
microglia	81
amyotrophic lateral sclerosis	80
mouse model	79

**Figure 7:**
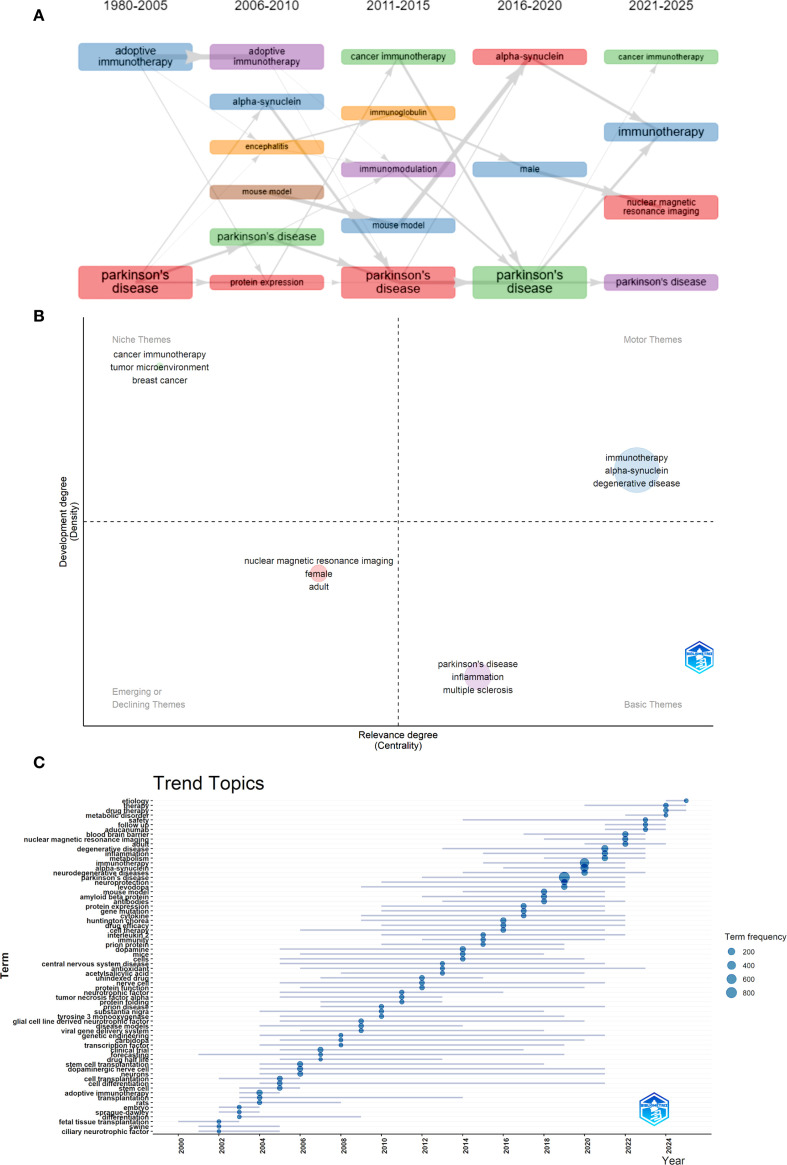
Hotspot and frontier analysis. **(A)** Thematic evolution. **(B)** Thematic Evolution in 2021-2025. **(C)** Trends Topics.

The Thematic Evolution function demonstrated the evolution process of key terms within the field. As time advanced, new themes emerged and interconnected, showing a shift toward more targeted and mechanistic studies. The rise of “alpha-synuclein” as a central research focus, alongside advancements in “adoptive immunotherapy,” “immunization,” and investigations into “microglia” and “mouse model” systems. In more recent years (2021–2025), the research scope expanded to include gender-specific considerations (“male” and “female”), cross-disease insights (“cancer immunotherapy”), and continued deep dives into the role of “alpha-synuclein” in Parkinson’s pathology (**Figures 7A, B**). The Topic Trends analysis also yielded similar results. Keywords related to precise immunotherapy intervention and efficacy assessment have become increasingly frequent since 2018. From the perspective of keyword duration, research on the immune mechanisms of PD and treatment technologies has exhibited a synergistic growth trend, reflecting the advancement of this field from pathogenesis analysis to clinical application and translation (**Figure 7C**).

### Clinical trials

Given that the keyword analysis indicates that clinical translation is currently the focus of research, we further utilized the PubMed database to search for relevant clinical trials in this field, providing additional references for researchers. Since the clinical study was not reviewed in accordance with the systematic review method, the summary of clinical trials is mainly presented in the supplementary materials. In total, eight clinical trials were searched and included (**Supplementary Table S10**; **Figure 1**). Tracing the registration number of the eight included clinical trials revealed that for six studies (7, 8, 16–19), the registered trial information was largely consistent with the designs reported in the original publications, indicating that these studies were conducted in strict accordance with their protocols and ensuring the reliability and consistency of the results. In contrast, two studies were registered in the EU CTR. Still, due to access limitations or a delay in updating by the investigator, the detailed trial protocols could not be fully retrieved (19, 20).

## Discussion

Through integrating the complementary resources of the WoSCC and Scopus databases, not only has the literature coverage been significantly expanded, but also the vigorous development potential and dynamic evolution characteristics of the PD immunotherapy field have been revealed. After merging the databases, the breadth of literature coverage and the depth of knowledge association have both improved significantly, suggesting that this field is currently in a critical stage of simultaneous expansion in academic output and influence. The complete timeline encompasses a higher average annual number of publications than a single database, further verifying the accumulated knowledge base and stable growth in research investment in this field.

The results of the journal analysis provide descriptive evidence of research activity and trends in the field of PD immunotherapy. The extensive coverage of 488 sources and the continuous increase in the number of publications in the top 10 journals directly reflect the expansion of research investment and the concentration of academic output in this field. The screening of core journals via Bradford’s Law reveals a concentration of publications in a subset of high-output journals, with journals that integrate neuroscience-focused and molecular biology-related content noted as prominent within the field. This distribution pattern aligns with the interdisciplinary characteristics of PD immunotherapy research.

At the author level, high-productivity scholars, such as Masliah E and Rockenstein E form a core influential group through their high publication volume and high H-index. Moreover, 85.96% of the authors have only published one paper, indicating that a large number of low-productivity authors limit the depth and sustainability of research. Notably, although researchers with high publication volumes and high H-indexes often form core, influential groups in a particular field, academic practices have also shown that there are cases where researchers have had multiple papers withdrawn due to suspected academic misconduct. This reminds us that the evaluation of research influence should take into account both academic output and research integrity, and avoid relying excessively on a single indicator.

Moreover, the University of California system and Harvard Medical School have a cooperation through institutions to enhance research output. The USA has the highest number of publications. Germany had the highest average article citations, but its average article citation and ranking dropped significantly after sequentially excluding review articles, extremely highly cited papers, or both, indicating that its high average citation mainly relied on special paper types such as reviews or extremely highly cited papers. The citation performance of countries/regions such as the USA, the United Kingdom, and China was relatively stable, especially the United Kingdom, which maintained the leading position in multiple analysis, followed by the USA, while China ranked in the middle to lower range but with minor fluctuations, suggesting that its citation indicators were not sensitive to reviews and extreme values. We further conducted a grouped analysis of the average article citations of various countries/regions by publication year period. Although the USA had fluctuations in rankings in individual stages, it generally ranked high. Overall, the citation performance of countries/regions changed over time, indicating that citation indicators have dynamic characteristics and need to be examined by time period to more comprehensively and scientifically evaluate the scientific research influence of each country. Notably, these metrics do not directly indicate differences in research quality or clinical translation potential. In addition, the USA and China have a lower proportion of international co-authorship compared to many other countries/regions. Despite the relatively low proportion of international cooperation among major countries like the USA and China, their large research volume and central roles in the network suggest potential for enhanced cooperation. However, the actual impact on clinical translation remains to be empirically validated.

Research in the field of PD immunotherapy has formed a highly interconnected core hotspot area within the current knowledge network. PD, as the central hub node of this network, is not only the focus of clinical research but also the convergence point of various pathological mechanisms and treatment strategies, reflecting its significant position in neurodegenerative diseases. Among them, nodes such as alpha-synuclein, neurodegeneration, and neuroinflammation reveal the key pathological processes of abnormal protein aggregation causing neuroinflammation and neuronal damage in PD, and these processes are precisely the important targets of current immunotherapy (21–26). At the same time, nodes related to the immune system, such as immune response, immune regulation, and immune system diseases, further highlight the therapeutic idea of regulating the immune microenvironment to slow disease progression. Moreover, nodes related to treatment methods, such as gene therapy, drug efficacy, and stem cell transplantation, have also been integrated into the research network, indicating that immunotherapy is not isolated but rather coexists with various emerging therapies to form part of a comprehensive intervention strategy for PD (27–29). Overall, this result reflects that current PD research is shifting from traditional symptomatic treatment to targeted immune regulation and multi-pathway combined treatment models. Immunotherapy, as a crucial bridge connecting neuroprotection, inflammation control, and disease modification, has become a key research direction and a potential breakthrough point in this field.

From a mechanistic perspective, α-synuclein, as the core research target, continues to make significant progress. The research on its association with microglia and neurodegenerative pathological processes is also deepening (30, 31). A recent study also indicates that in mice injected with α-synuclein collagen, reducing the activity of microglia can slow down the spread of pathological α-synuclein lesions (32). In terms of treatment strategies, precise immunological intervention has become the trend. Technologies such as adoptive immunotherapy and immunization are constantly emerging, driving the translation from basic research to clinical application. For example, the targeted delivery system mediated by nanocarriers fosters the development of precise treatment (33). Meanwhile, cross-dimensional research has expanded the frontier boundaries, and gender-specific research incorporates perspectives of differences between men and women, providing possibilities for personalized treatment (34).

Currently, clinical research on immunotherapy for PD remains in the early validation stage, with most studies focusing on active immunotherapy, such as PD01A and UB-312, which aim to regulate α-synuclein aggregation or restore the dopaminergic system (7, 20). Volc et al. reported that PD01A induced a significant humoral immune response, binding to the intended target; however, the study was limited by its non-randomized design and small sample size (20). Similarly, Poewe et al. demonstrated that PD03A elicited antibody responses with acceptable safety, supporting further development of active immunotherapy, but also highlighted the need for long-term follow-up (35). UB-312 was initially tested in healthy participants, showing good safety and the induction of anti-α-synuclein (8). A subsequent phase revealed that reduced α-synuclein core structures in cerebrospinal fluid, although without significant clinical improvement. Other approaches have included Lu AF82422, which was shown to be safe and pharmacokinetically suitable for further development (8). Moreover, Olson et al. demonstrated that sargramostim was safe and could restore immune balance, but in a small sample. In addition, trials with the monoclonal antibody BIIB054 in both healthy participants and PD patients consistently confirmed favorable safety and target engagement, although these findings were again limited by small cohorts (16, 17, 19). Overall, these trials suggest that active immunotherapy and targeted antibodies generally demonstrate favorable safety and immunogenicity in PD.

However, most trials were exploratory, involved small sample sizes, and often relied on non-randomized designs. Furthermore, they also lacked long-term follow-up data. For example, some studies have focused only on immune responses rather than clinical symptoms, and have shown limited population diversity, with many studies restricted to healthy participants or Western cohorts (7, 8, 19). These limitations reduce the reliability of conclusions regarding efficacy and safety. Looking ahead, large-scale randomized controlled trials involving diverse populations, long-term follow-up, and dynamic biomarker monitoring are needed to verify the stability of therapeutic effects. New biomarkers based on cerebrospinal fluid have been discovered, and they may gradually influence the overall design of clinical intervention trials. Identifying biomarkers for PD may enable earlier and more accurate diagnosis and treatment (36–38).

Comparison of the registered protocols with the actual studies revealed that most trials were conducted mainly in accordance with their initial registrations, with only minor modifications observed. For example, in the study by Brys, the registered duration was 20 weeks, whereas the actual trial lasted 16 weeks (20). Additional cohorts (135-mg/kg HV and early PD groups) were introduced after trial initiation, based on safety, tolerability, pharmacokinetic, and adverse event considerations, reflecting a participant-centered approach with timely adjustments to ensure safety and feasibility. Interestingly, one of the studies represented a secondary analysis of samples from previous clinical trials (19). It aimed to develop a zero-length crosslinking method, combined with Meso Scale Discovery technology, to enable the quantitative analysis of cinpanemab-α-synuclein complexes in clinical cerebrospinal fluid samples by preventing signal loss due to rapid dissociation. This work not only highlights the challenges and resource demands of clinical trials but also emphasizes the significance of methodological innovation. Applying fundamental research techniques, such as chemical crosslinking, directly to clinical samples addresses practical issues in drug development and disease diagnosis. Such secondary analyses, or “*post-hoc* dissections” of completed trials, provide critical insights for subsequent development and create a translational feedback loop from clinic to laboratory and back, representing an innovative and resource-efficient research strategy.

## Limitations

Our study has some limitations. Firstly, the bibliometric indicators are difficult to capture the heterogeneity of research quality. For instance, the achievements of prolific authors may include low-impact conference papers, while breakthrough studies by less productive authors may not have been adequately measured. Importantly, when assessing the influence of scientific research, it is essential to consider not only academic output indicators but also the credibility and academic ethics of the research. Future research can further explore how to incorporate indicators such as academic integrity records and research reproducibility into the comprehensive evaluation system. Secondly, although keyword co-occurrence analysis can map research hotspots, it cannot reveal the implicit interdisciplinary connections, and it relies on the standardized use of terms by researchers. Furthermore, we are unable to obtain the unique identifiers for all authors. Some author nodes may have duplication, incorrect merging or splitting issues (39).Visualization analysis only reflects the co-occurrence intensity and cannot verify the causal relationship of mechanisms, nor can it provide biological evidence (40, 41). Finally, due to the lack of pre-established strict criteria for including intervention types and relying on the descriptions of intervention measures in the literature, there is a potential misclassification risk in the current summary of clinical trials.

## Conclusions

The present bibliometric analysis shows that PD immunotherapy is a rapidly expanding field. The extensive coverage of 488 sources and the continuous increase in the number of publications in the top 10 journals directly reflect the expansion of research investment and the concentration of academic output in this field. A large number of low-productivity authors limit the depth and sustainability of research. The USA has the highest number of publications. The citation indicators among different countries/regions change over time and exhibit dynamic characteristics. Additionally, the USA and China have a lower proportion of international co-authorship compared to many other countries or regions.

The key pathological processes, such as neuroinflammation and neuronal damage caused by the abnormal aggregation of α-synuclein, as well as therapeutic ideas for regulating the immune microenvironment to delay disease progression, are current research hotspots. Precise immunological intervention techniques, such as adoptive immunotherapy and targeted delivery via nanocarriers, are continually emerging, promoting the translation of basic research into clinical applications, and are frontiers in this field. While clinical trials remain exploratory and limited in scale, bibliometric evidence suggests an increasing interest in bridging the gap between basic and clinical research. Future progress will require large-scale, collaborative studies with robust designs and dynamic biomarker monitoring, as well as exploration of combination strategies, to strengthen clinical translation and advance personalized immunotherapy for PD.

## Data Availability

The original contributions presented in the study are included in the article/**Supplementary Material**. Further inquiries can be directed to the corresponding authors.
