# Supplementary material for: Research on Parkinson’s disease immunotherapy: a bibliometric analysis via multiple databases
Source: Front Immunol. 2025 Oct 8;16:1659848. doi: 10.3389/fimmu.2025.1659848 (PMC12547164; doi:10.3389/fimmu.2025.1659848)
Supplement: Supplementary file 1 [file DataSheet1.pdf]

Table S1 Search strategy via Web of Science Core Collection

Step	Search strategy
#1	TS= ("immunotherapy" OR "immunotherapies" OR "immunotherapeutic" OR"immunotherapeutics" OR "immunotherap*")
#2	TS= (“Parkinson's disease” OR “Parkinson disease” OR “Parkinson's diseas*” OR “Parkinson diseas*” OR “Idiopathic Parkinson Disease” OR “Idiopathic Parkinson's Disease” OR “Idiopathic Parkinson Diseas*” OR “Idiopathic Parkinson's Diseas*” OR “Lewy Body Parkinson's Disease” OR “Lewy Body Parkinson Disease” OR “Lewy Body Parkinson's Diseas*” OR “Lewy Body Parkinson Diseas*” OR “Paralysis Agitans” OR “Paralysis Agitan*” OR “Primary Parkinsonism” OR “Primary Parkinsonis*”)
#3	#1 AND #2

Table S2 Search strategy via Scopus

Step	Search strategy
#1	((TITLE-ABS-KEY ("Parkinson's disease") OR TITLE-ABS-KEY ("Parkinson disease") OR TITLE-ABS-KEY ("Parkinson's diseas*") OR TITLE-ABS-KEY ("Parkinson diseas*") OR TITLE-ABS-KEY ("Idiopathic Parkinson Disease") OR TITLE-ABS-KEY ("Idiopathic Parkinson's Disease") OR TITLE-ABS-KEY ("Idiopathic Parkinson Diseas*") OR TITLE-ABS-KEY ("Idiopathic Parkinson's Diseas*") OR TITLE-ABS-KEY ("Lewy Body Parkinson's Disease") OR TITLE-ABS-KEY ("Lewy Body Parkinson Disease") OR TITLE-ABS-KEY ("Lewy Body Parkinson's Diseas*") OR TITLE-ABS-KEY ("Lewy Body Parkinson Diseas*") OR TITLE-ABS-KEY ("Paralysis Agitans") OR TITLE-ABS-KEY ("Paralysis Agitan*") OR TITLE-ABS-KEY ("Primary Parkinsonism") OR TITLE-ABS-KEY ("Primary Parkinsonis*"))) AND ((TITLE-ABS-KEY (immunotherapy) OR TITLE-ABS-KEY (immunotherapies) OR TITLE-ABS-KEY (immunotherapeutic) OR TITLE-ABS-KEY (immunotherapeutics) OR TITLE-ABS-KEY (immunotherap*)))

Table S3 Search strategy via PubMed

Step	Search strategy
#1	"Parkinson Disease"[Mesh] OR "Parkinson's disease"[Title/Abstract] OR "Parkinson disease"[Title/Abstract] OR "Parkinson's diseas*"[Title/Abstract] OR "Parkinson diseas*"[Title/Abstract] OR "Idiopathic Parkinson Disease"[Title/Abstract] OR "Idiopathic Parkinson's Disease"[Title/Abstract] OR "Idiopathic Parkinson Diseas*"[Title/Abstract] OR "Idiopathic Parkinson's Diseas*"[Title/Abstract] OR "Lewy Body Parkinson's Disease"[Title/Abstract] OR "Lewy Body Parkinson Disease"[Title/Abstract] OR "Lewy Body Parkinson's Diseas*"[Title/Abstract] OR "Lewy Body Parkinson Diseas*"[Title/Abstract] OR "Paralysis Agitans"[Title/Abstract] OR "Paralysis Agitan*"[Title/Abstract] OR "Primary Parkinsonism"[Title/Abstract] OR "Primary Parkinsonis*"[Title/Abstract]
#2	"Immunotherapy"[Mesh] OR "immunotherapy"[Title/Abstract] OR "immunotherapies"[Title/Abstract] OR "immunotherapeutic" OR"immunotherapeutics"[Title/Abstract] OR "immunotherap*"[Title/Abstract]
#3	#1 AND #2

Table S4 Countries/regions citation indicators

Country/Region	General statistics			Sensitivity analysis								
				Exclude reviews			Exclude top 5% highly cited papers			Exclude reviews and top 5% highly cited papers		
	Rank	Citation	Average Article Citation	Rank	Citation	Average Article Citation	Rank	Citation	Average Article Citation	Rank	Citation	Average Article Citation
Germany	1	5202	179.4	7	208	16.0	8	326	12.5	7	208	16.0
USA	2	13124	56.6	1	5218	47.4	2	6118	28.6	3	2073	20.9
United Kingdom	3	2808	49.3	2	633	27.5	1	1582	29.3	1	633	27.5
Spain	4	892	40.5	4	141	17.6	3	557	26.5	4	141	17.6
Italy	5	1638	33.4	3	366	21.5	5	865	18.8	2	366	21.5
Japan	6	350	19.4	5	122	17.4	4	350	19.4	5	122	17.4
Canada	7	484	17.9	10	92	7.7	6	484	17.9	10	92	7.7
China	8	1624	15.0	9	500	9.4	7	1624	15.0	9	500	9.4
India	9	673	15	6	104	17.3	9	483	11.0	6	104	17.3
Iran	10	172	9.6	8	59	9.8	10	172	9.6	8	59	9.8

Table S5 Country/region citation analysis grouped by publication year

Country/Region	General statistics			Publication year cohorts								
				1980-2005			2006-2015			2016-2025		
	Rank	Citation	Average Article Citation	Rank	Citation	Average Article Citation	Rank	Citation	Average Article Citation	Rank	Citation	Average Article Citation
Germany	1	5202	179.4	8	8	4.0	1	4727	675.3	3	467	23.4
USA	2	13124	56.6	2	2484	88.7	6	2942	47.5	2	7698	54.2
United Kingdom	3	2808	49.3	5	200	16.7	5	523	52.3	1	2085	59.6
Spain	4	892	40.5	1	488	122.0	8	116	23.2	4	288	22.2
Italy	5	1638	33.4	3	31	31.0	2	1144	76.3	6	463	14.0
Japan	6	350	19.4	4	90	30.0	7	119	39.7	8	141	11.8
Canada	7	484	17.9	7	18	4.5	4	219	54.8	7	247	13.0
China	8	1624	15.00	6	13	6.5	9	97	8.8	5	1514	15.9
India	9	673	15	9	0	0.0	3	334	55.7	10	339	8.9
Iran	10	172	9.6	10	0	0.0	10	3	3.0	9	169	9.9

Table S6 Cooperation analysis of authors

Author	Cluster	Betweenness	Closeness	PageRank
Wang Y	1	150.27	0.01	0.04
Liu Y	1	1.14	0.01	0.01
Zhang J	1	20.36	0.01	0.02
Wang J	1	7.55	0.01	0.02
Li X	1	60.29	0.01	0.02
Chen S	1	6.43	0.01	0.02
Chen Y	1	2.33	0.01	0.02
Liu C	1	1.83	0.01	0.01
Wang X	1	0.00	0.01	0.01
Liu J	1	46.26	0.01	0.02
Yang H	1	20.50	0.01	0.02
Zhang X	1	37.01	0.01	0.02
Zhao Y	1	0.00	0.01	0.01
Brundin P	2	3.00	0.20	0.02
Kordower J	2	0.00	0.13	0.01
Mcfarthing K	2	0.00	0.14	0.02
Stott S	2	1.00	0.20	0.03
Wyse R	2	1.00	0.20	0.03
Gendelman H	3	0.00	1.00	0.02
Mosley R	3	0.00	1.00	0.02
Masliah E	4	95.85	0.01	0.05
Rockenstein E	4	10.90	0.01	0.04
Adame A	4	8.47	0.01	0.04
Lee S	4	199.85	0.01	0.03
El-Agnaf O	4	69.74	0.01	0.02
Mante M	4	5.11	0.01	0.03
Spencer B	4	1.85	0.01	0.03
Kim C	4	3.62	0.01	0.03
Overk C	4	2.54	0.01	0.03
Schneeberger A	4	19.07	0.01	0.02
Mandler M	4	0.01	0.01	0.01
Rissman R	4	3.77	0.01	0.03
Vaikath N	4	69.43	0.01	0.02
Valera E	4	0.00	0.01	0.02

Iba M	4	0.00	0.01	0.01
Jankovic J	4	0.00	0.01	0.00
Kim J	4	25.59	0.01	0.01
Lee J	4	101.59	0.01	0.01
Li J	4	177.96	0.01	0.01
Majbour N	4	31.27	0.01	0.02
Poewe W	4	37.00	0.01	0.01
Ingelsson M	5	88.58	0.01	0.02
Bergström J	5	22.82	0.01	0.02
Lannfelt L	5	0.00	0.01	0.02
Möller C	5	0.00	0.01	0.02
Nordström E	5	0.00	0.01	0.02

Table S7 Cooperation analysis of institutions

Institution	Cluster	Betweenness	Closeness	PageRank
Harvard University	1	75.94	0.01	0.05
Harvard Medical School	1	141.30	0.01	0.05
University of California	1	0.00	0.01	0.00
University of Nebraska Medical Center	1	0.00	0.01	0.00
Columbia University	1	1.90	0.01	0.01
Harvard University Medical Affiliates	1	3.88	0.01	0.03
University of British Columbia	1	41.20	0.01	0.02
Hebrew University of Jerusalem	1	38.00	0.01	0.01
Laval University	1	0.00	0.01	0.01
Mcgill University	1	90.91	0.01	0.02
Baylor College of Medicine	2	38.00	0.01	0.02
National University of Singapore	2	0.00	0.01	0.01
University of Texas System	3	38.00	0.01	0.02
University of Texas Medical Branch Galveston	3	0.00	0.01	0.02
University of California System	4	173.87	0.01	0.06
University of California San Diego	4	6.47	0.01	0.04
University of Cambridge	4	64.19	0.01	0.02
National Institutes of Health (NIH) - Usa	4	65.09	0.01	0.05
NIH National Institute on Aging (NIA)	4	41.82	0.01	0.05
Qatar Foundation (QF)	4	13.40	0.01	0.03
University Of Oxford	4	13.72	0.01	0.02
Hamad Bin Khalifa University-Qatar	4	13.40	0.01	0.03
Seoul National University (SNU)	4	3.22	0.01	0.02
Lund University	4	0.83	0.01	0.01
Qatar Biomedical Research Institute (QBRI)	4	3.68	0.01	0.02
State University of New York (SUNY) System	4	0.00	0.01	0.01
University College London	4	0.00	0.01	0.01
University of Ottawa	5	91.13	0.01	0.02
Van Andel Institute	5	21.50	0.01	0.01
Aarhus University	6	4.63	0.01	0.01
University of Copenhagen	6	12.68	0.01	0.01
University of Toronto	7	24.90	0.01	0.02
Uppsala University	7	0.00	0.01	0.02
Eberhard Karls University of Tubingen	7	5.33	0.01	0.03

University of Pennsylvania	7	37.01	0.01	0.03
Eberhard Karls University Hospital	7	5.33	0.01	0.03
German Center for Neurodegenerative Diseases (DZNE)	7	5.33	0.01	0.04
Helmholtz Association	7	5.33	0.01	0.04
Centre National De La Recherche Scientifique (CNRS)	7	0.00	0.01	0.01
Capital Medical University	8	0.00	1.00	0.02
Central South University	8	0.00	1.00	0.02
State University System of Florida	9	1.00	0.50	0.03
University of Florida	10	0.00	0.33	0.02
University of South Florida	11	0.00	0.33	0.01
Tel Aviv University	12	0.00	0.01	0.01

Table S8 Cooperation analysis of countries/regions

Node	Cluster	Betweenness	Closeness	PageRank
USA	1	184.02	0.02	0.14
Germany	1	39.81	0.02	0.05
Italy	1	39.14	0.01	0.03
Canada	1	14.73	0.02	0.05
Iran	1	1.29	0.01	0.01
Spain	1	1.48	0.01	0.02
Sweden	1	26.62	0.02	0.05
Israel	1	0.00	0.01	0.01
Denmark	1	0.27	0.01	0.01
Netherlands	1	5.64	0.01	0.03
Mexico	1	6.26	0.01	0.01
United Kingdom	2	189.64	0.02	0.11
France	2	1.37	0.01	0.03
Austria	2	2.69	0.01	0.03
Singapore	2	0.17	0.01	0.01
Switzerland	2	14.52	0.01	0.02
Hungary	2	0.17	0.01	0.02
Ireland	2	0.00	0.01	0.01
Korea	3	0.15	0.01	0.02
Australia	3	0.24	0.01	0.02
Qatar	3	39.40	0.02	0.03
Greece	3	6.64	0.01	0.02
U Arab Emirates	3	1.47	0.01	0.02
China	4	54.31	0.02	0.05
Japan	4	17.25	0.02	0.03
South Africa	5	0.00	0.01	0.01
Iraq	5	0.00	0.01	0.01
Jordan	5	0.00	0.01	0.01
Egypt	6	0.00	0.01	0.01
Poland	6	0.00	0.01	0.01
Uganda	6	0.00	0.01	0.01
India	7	1.93	0.01	0.01
Malaysia	8	0.00	0.01	0.01
Portugal	9	0.00	0.01	0.01

Saudi Arabia	10	112.78	0.01	0.03
Russia	11	0.00	0.01	0.01
Finland	12	0.00	0.01	0.01
Lebanon	13	0.00	0.01	0.01

Table S9 Keyword co-occurrence analysis

Keyword	Cluster	Betweenness	Closeness	PageRank
levodopa	1	0.536	0.02	0.018
adoptive immunotherapy	1	0.168	0.018	0.014
dopamine	1	0.383	0.02	0.016
clinical trial	1	0.352	0.02	0.014
protein expression	1	0.406	0.02	0.016
gene expression	1	0.303	0.02	0.014
male	1	0.27	0.019	0.012
nuclear magnetic resonance imaging	1	0.2	0.019	0.012
stem cell transplantation	1	0.188	0.019	0.013
controlled study	1	0.416	0.019	0.013
dopaminergic nerve cell	1	0.285	0.019	0.014
neurologic disease	1	0.196	0.02	0.012
female	1	0.1	0.017	0.011
parkinson's disease	2	33.661	0.02	0.098
immunotherapy	2	13.513	0.02	0.074
alpha-synuclein	2	3.337	0.02	0.038
degenerative disease	2	1.531	0.02	0.037
neuroprotection	2	0.783	0.02	0.026
neurodegenerative diseases	2	0.584	0.02	0.024
inflammation	2	0.269	0.019	0.02
metabolism	2	0.568	0.02	0.023
neurodegeneration	2	0.303	0.019	0.016
pathology	2	0.595	0.02	0.018
cancer immunotherapy	2	0.188	0.019	0.014
multiple sclerosis	2	0.387	0.02	0.02
gene therapy	2	0.229	0.019	0.016
nerve degeneration	2	0.48	0.02	0.025
nervous system inflammation	2	0.313	0.02	0.023
microglia	2	0.436	0.02	0.021
amyotrophic lateral sclerosis	2	0.364	0.02	0.02
mouse model	2	0.027	0.014	0.009
blood brain barrier	2	0.357	0.02	0.02
huntington chorea	2	0.338	0.02	0.019
oxidative stress	2	0.356	0.02	0.02

brain	2	0.314	0.02	0.016
immune response	2	0.379	0.02	0.017
pathogenesis	2	0.221	0.02	0.016
amyloid beta protein	2	0.276	0.02	0.019
dementia	2	0.226	0.019	0.014
protein aggregation	2	0.292	0.02	0.018
signal transduction	2	0.232	0.02	0.016
immunology	2	0.249	0.02	0.016
immunization	2	0.257	0.018	0.012
genetics	2	0.164	0.019	0.014
central nervous system	2	0.231	0.02	0.014
drug efficacy	2	0.165	0.02	0.013
apoptosis	2	0.097	0.019	0.014
monoclonal antibody	2	0.114	0.019	0.013
neurotoxicity	2	0.218	0.02	0.015
procedures	2	0.14	0.02	0.013

Table S10 Summary of Clinical trials in PD immunotherapy field*

First-Author	Year	Participants	Treatment	Effects	Study limitations	Trial registration	Reference
Dieter Volc	2020	24	PD01A	The specific active immunotherapy triggered a significant humoral immune response and achieved binding to the target.	Non-randomized controlled study; A phase II study is needed to further evaluate the safety and efficacy of PD01A in the treatment of Parkinson's disease.	EudraCT numbers: 2011–002650–31, 2013–001774–20, 2014–002489–54, and 2015–004854–16	(1)
Katherine E Olson	2021	5	Sargramostim	Long-term treatment with sargramostim at a dose of 3 micrograms per kilogram per day is safe and effective, and it can restore immune balance.	Non-randomized controlled study; Small sample size; A larger scale of patient samples is needed for assessment to determine the efficacy of the drug	ClinicalTrials.gov: NCT03790670	(2)
Miroslaw Brys	2019	66 (48 healthy volunteers + 18 Parkinson's disease participants)	BIIB054	BIIB054 exhibits excellent safety, tolerability, and pharmacokinetic characteristics in both volunteers and patients with Parkinson's disease.	Small sample size	ClinicalTrials.gov: NCT02459886	(3)
Hui Jing Yu	2022	50 healthy volunteers	UB-312	UB-312 generally has good safety and excellent tolerance, and it has induced the production of anti-α-synuclein antibodies in the serum and cerebrospinal fluid of healthy participants	Non-randomized controlled study; only studied healthy subjects	ClinicalTrials.gov: NCT04075318	(4)
Werner Poewe	2021	36 (PD03A 15μg: 12, PD03A 75μg: 12; placebo: 12)	PD03A	The safety features of PD03A and the resulting positive antibody responses have supported the further development of active	Small sample size; there is a lack of long-term exploration.	EudraCT: 2014-000568-16	(5)

				immunotherapy approaches targeting PD.			
Pepijn Eijsvogel	2024	20 (UB-312 300/100/100 µg: 7; UB-312 300/300/300 µg: 7; Placebo: 6)	UB-312	For some patients who received treatment with UB-312, there was a significant reduction in the core structure of α-synuclein in their cerebrospinal fluid.	Small sample size; there was no statistically significant difference in the clinical scores.	ClinicalTrials.gov : NCT04075318	(6)
Louise Buur	2024	73 (58 healthy volunteers + 15 Parkinson's disease participants)	Lu AF82422	The safety and pharmacokinetic characteristics of Lu AF82422 are suitable for further clinical development. The results indicate that it can act on peripheral targets.	Small sample size	ClinicalTrials.gov : NCT03611569	(7)
YuTing Liu	2025	64 (46 healthy volunteers + 18 Parkinson's disease participants)	BIIB054	Dose- and time-dependent binding are consistent with cinpanemab's affinity for α-synuclein and provided confidence that the drug had engaged its target at the desired site of action.	Small sample size	ClinicalTrials.gov : NCT02459886	(8)

*The clinical trials are obtained from PubMed, and the search process for these data does not fully comply with the requirements of a systematic review. **PD:** Parkinson's disease.

References

1. Volc D, Poewe W, Kutzelnigg A, Lührs P, Thun-Hohenstein C, Schneeberger A, Galabova G, Majbour N, Vaikath N, El-Agnaf O, Winter D, Mihailovska E, Mairhofer A, Schwenke C, Staffler G, Medori R. Safety and immunogenicity of the α-synuclein active immunotherapeutic PD01A in patients with Parkinson's disease: a randomised, single-blinded, phase 1 trial. The Lancet Neurology. 2020 Jul;19(7):591-600. eng. Epub 2020/06/21. doi:10.1016/s1474-4422(20)30136-8. Cited in: Pubmed; PMID 32562684.
2. Olson KE, Namminga KL, Lu Y, Schwab AD, Thurston MJ, Abdelmoaty MM, Kumar V, Wojtkiewicz M, Obaro H, Santamaria P, Mosley RL, Gendelman HE. Safety, tolerability, and immune-biomarker profiling for year-long sargramostim treatment of Parkinson's disease. EBioMedicine. 2021 May;67:103380. eng. Epub 2021/05/18. doi:10.1016/j.ebiom.2021.103380. Cited in: Pubmed; PMID 34000620.
3. Brys M, Fanning L, Hung S, Ellenbogen A, Penner N, Yang M, Welch M, Koenig E, David E, Fox T, Makh S, Aldred J, Goodman I, Pepinsky B, Liu Y, Graham D, Weihofen A, Cedarbaum JM. Randomized phase I clinical trial of anti-α-synuclein antibody BIIB054. Movement disorders : official journal of the Movement Disorder Society. 2019 Aug;34(8):1154-1163. eng. Epub 2019/06/19. doi:10.1002/mds.27738. Cited in: Pubmed; PMID 31211448.
4. Yu HJ, Thijssen E, van Brummelen E, van der Plas JL, Radanovic I, Moerland M, Hsieh E, Groeneveld GJ, Dodart JC. A Randomized First-in-Human Study With UB-312, a UBITh® α-Synuclein Peptide Vaccine. Movement disorders : official journal of the Movement Disorder Society. 2022 Jul;37(7):1416-1424. eng. Epub 2022/04/16. doi:10.1002/mds.29016. Cited in: Pubmed; PMID 35426173.
5. Poewe W, Volc D, Seppi K, Medori R, Lührs P, Kutzelnigg A, Djamshidian A, Thun-Hohenstein C, Meissner WG, Rascol O, Schneeberger A, Staffler G, Poewe W, Seppi K, Djamshidian A, deMarzi R, Heim B, Mangesius S, Stolz R, Wachowicz K, Volc D, Thun-Hohenstein C, Riha C, Schneeberger A, Bürger V, Galabova G. Safety and Tolerability of Active Immunotherapy Targeting α-Synuclein with PD03A in Patients with Early Parkinson's Disease: A Randomized, Placebo-Controlled, Phase 1 Study. Journal of Parkinson's disease. 2021;11(3):1079-1089. eng. Epub 2021/06/08. doi:10.3233/jpd-212594. Cited in: Pubmed; PMID 34092654.
6. Eijsvogel P, Misra P, Concha-Marambio L, Boyd JD, Ding S, Fedor L, Hsieh YT, Sun YS, Vroom MM, Farris CM, Ma Y, de Kam ML, Radanovic I, Vissers M, Mirski D, Shareghi G, Shahnawaz M, Singer W, Kremer P, Groeneveld GJ, Yu HJ, Dodart JC. Target engagement and immunogenicity of an active immunotherapeutic targeting pathological α-synuclein: a phase 1 placebo-controlled trial. Nature medicine. 2024 Sep;30(9):2631-2640. eng. Epub 2024/06/21. doi:10.1038/s41591-024-03101-8. Cited in: Pubmed; PMID 38902546.
7. Buur L, Wiedemann J, Larsen F, Ben Alaya-Fourati F, Kallunki P, Ditlevsen DK, Sørensen MH, Meulien D. Randomized Phase I Trial of the α-Synuclein Antibody Lu AF82422. Movement disorders : official journal of the Movement Disorder Society. 2024 Jun;39(6):936-944. eng. Epub 2024/03/18. doi:10.1002/mds.29784. Cited in: Pubmed; PMID 38494847.
8. Liu Y, Yang M, Fraser K, Graham D, Weinreb PH, Weihofen A, Hirst WD, Cedarbaum JM, Pepinsky B. Quantification of cinpanemab (BIIB054) binding to α-synuclein in cerebrospinal fluid of phase 1 single ascending dose samples. The Journal of pharmacology and experimental therapeutics. 2025 Jan;392(1):100003. eng. Epub 2025/02/02. doi:10.1124/jpet.124.002199. Cited in: Pubmed; PMID 39892989.
